# Effect of clarithromycin in patients with Gram-negative sepsis: subgroup analysis of a randomized trial

**DOI:** 10.1186/cc13432

**Published:** 2014-03-17

**Authors:** E Giamarellos-Bourboulis, K Lymberopoulou, I Tsangaris, A Antonopoulou, A Marioli, L Leonidou, E Douzinas, I Koutelidakis, A Armaganidis

**Affiliations:** 1 University of Athens, Medical School, Athens, Greece; 2 Sismanogleion General Hospital, Athens, Greece; 3 University of Athens, Athens, Greece; 4 University of Patras, Rion, Greece; 5 Aristotle University, Thessaloniki, Greece

## Introduction

A recent randomized trial of our group showed that blind treatment with clarithromycin decreased mortality from septic shock and multiple organ dysfunction, shortened time until resolution of infection in patients with severe sepsis/shock and decreased hospitalization costs [1]. The efficacy of clarithromycin in relation with the type of failing organs is analyzed.

## Methods

Six hundred patients with systemic inflammatory response syndrome due to primary Gram-negative bacteremia or acute pyelonephritis or intraabdominal infection were blindly assigned to placebo or clarithromycin for four consecutive days as adjunctive treatment to standard of care. Clarithromycin was administered at a dose of 1 g once daily in 1 hour of continuous infusion. Organ failures before allocation to blind treatment were defined according to the Surviving Sepsis Campaign 2003 definitions. Cox regression analysis was done to verify the effect of clarithromycin as a moderator. Hazard ratios (HRs) and 95% confidence intervals (CIs) were calculated.

## Results

Forty-nine patients of the placebo arm and 55 patients of the clarithromycin arm had acute lung injury (ALI); mortality was 51.0% and 30.9% respectively (*P *= 0.046). Forty-seven patients of the placebo arm and 54 patients of the clarithromycin arm had acute coagulopathy; mortality was 44.7% and 44.4% respectively (*P *= 1.000). Twenty patients of the placebo arm and 19 patients of the clarithromycin arm had metabolic acidosis; mortality was 55.0% and 52.6% respectively (*P *= 1.000). Twenty-nine patients of the placebo arm and 39 patients of the clarithromycin arm had acute oliguria; mortality was 55.2% and 48.7% respectively (*P *= 0.631). ALI (HR = 2.42; 95% CI = 1.45 to 4.03, *P *= 0.001), acute coagulopathy (HR = 2.65; CI = 1.69 to 4.16) and cardiovascular failure (HR = 3.36, CI = 2.09 to 5.39) were independently associated with unfavorable outcome. Adding treatment with clarithromycin in the equation reduced the risk for death by ALI by 1.86-fold (HR = 0.54; CI = 0.29 to 0.99, *P *= 0.049).

## Conclusion

Clarithromycin is a major moderator of the physical course of Gram-negative sepsis complicated with ALI.

## References

[B1] Giamarellos-Bourboulis EK J Antimicrob Chemother 2013

